# Return on Investment (ROI) for Continuous Research and Development (R&D) Funding for Innovation‐Driven Medical Research: From Knowledge to Policy

**DOI:** 10.1155/bmri/2544260

**Published:** 2026-04-27

**Authors:** Seyed Shahmy, Sirimali Fernando, Sirimal Abeyratne, Ajith De Alwis

**Affiliations:** ^1^ National Science and Technology Commission of Sri Lanka, Battaramulla, Sri Lanka; ^2^ Faculty of Graduate Studies, University of Moratuwa, Moratuwa, Sri Lanka, mrt.ac.lk; ^3^ Department of Microbiology, Faculty of Medical Sciences, University of Sri Jayewardenepura, Nugegoda, Sri Lanka, sjp.ac.lk; ^4^ Department of Economics, Faculty of Arts, University of Colombo, Colombo, Sri Lanka, cmb.ac.lk

Global spending on health is $9.9 trillion, which is about 9.9% of global GDP in 2022, a decline from 2021 in real terms (10.4%), but it is projected to rise 10.7% in 2023, including the significant allocations to research and development (R&D) activities in the sector [1]. Innovation‐driven medical research is crucial since it can lead to new treatments and improved patient care, thereby resulting in better healthcare outcomes. However, the nations in the global south frequently failed to consider R&D expenditures as investments from a socioeconomic perspective, which led to policymakers failing to pay adequate attention to ensure consistent and sufficient funding. Occasionally, a scientific breakthrough saves the lives of millions of people, creates new opportunities for society, and points communities in the direction of a prosperous future. Nevertheless, it is a well‐known fact that 90% of the medical research, in particular to bring a new treatment—pharmaceuticals for human use—ends up with negative results that seem to show no immediate monetary benefits to the economy. But this grey phase in the innovation value creation chain—the “Death Valley”—is not fully captured by most of the economic models, such as to reflect the long‐term effects of the knowledge incubation, accumulation, and spillovers within the context of social capital shocks [2].

In general, there is a strong correlation between the development of a country and its investment in R&D; therefore, the investments in innovation‐driven science and technology (S&T) are frequently considered a source of national prestige by the nations [3]. A substantial amount of expenditure by nations on R&D is directed towards public‐sector universities and research institutions—for the purposes of knowledge generation, human capital development, and infrastructure—thereby anticipating a significant contribution to the national economy. Assessing the returns on them requires a comprehensive socioeconomic framework, given the inherent complexity of the various contextual dimensions in economic, health, and social aspects. Within these dimensions, it involves creating new knowledge, practices, technologies, skills, and expertise; enhancing the value of national knowledge assets; creating jobs as a result of spinoffs and diffusion and spillovers; and ultimately strengthening the national purchasing power in terms of the standard of living of the society that would have all the potential to sustain the funding for R&D [4,5].

The endogenous economic growth model is the first to elucidate the effects of knowledge generation on the economy regarding the return on investment (ROI) in R&D. In this model, knowledge, as a nonrival form of human capital, along with technological innovations, serves as a crucial parameter for sustainable long‐term economic growth [6]. Accordingly, several studies have been explored to assess the ROI in R&D, and one of such landmark studies—believed to be a gold standard study—calculated the ROI in the US genome study. The study estimated that $1 invested in the US Genome project between 1990 and 2003 generated $141 and continued to do so [7].

In the digitalization era, knowledge is no longer a nonrival since the high‐skilled labour—knowledge accumulation—has a high possibility to flow horizontally in the “global cloud village”. In particular, in the discipline of medical sciences and health care, due to rapid changes in the discipline driven by technological advancements, research breakthroughs, and evolving societal needs, it might be highly likely. The scenario calls for drawing a semiendogenous complete model in which human capital—skills and education, ability, and the speed and accessibility of knowledge transfer—can all act together to provide vast opportunities that will improve the performance of the R&D sector [8]. The inclusivity of the capital—which is made up of social, intellectual, and structural dimensions as a matrix along with technological innovation, knowledge, and human capital as parameters (Figure S1)—allows the model to construct an adjusted traditional production function [9, 10]. The possible modification in the model is expected to draw the information to strengthen the informed decision‐making—an effective science brokering to ensure the continuous R&D funds at the science and policy interface, in particular innovation‐driven medical research—knowledge to policy—in pandemics, noncommunicable diseases, health inequality, pollution, the Internet of medical things, rehabilitation, food security and nutrients, ageing population, patient experience, and climate change, to name a few [11–15].

In essence, inadequate funding for R&D can lead to inconclusive research outcomes and a failure to produce unbiased knowledge that supports the evidence. This limitation hinders knowledge brokering, which struggles to comprehend how funds are allocated concerning ROI. Consequently, it widens the gap in informed decision‐making at the science–policy interface and ultimately results in a depletion of future funds (Figure S1).

Are we ready to embrace this? Do we have a futuristic plan to tackle this? There are no absolute answers for either.

## Funding

No funding was received for this manuscript.

## Disclosure

The initial version of this article, which has not undergone peer review, was previously published as a preprint on Authorea and ResearchGate [14]. The views expressed in the article are purely those of the writers and may not under any circumstances be regarded as stating the official position of their affiliated institutions.

## Conflicts of Interest

The authors declare no conflicts of interest.

## Supporting information


**Supporting Information** Additional supporting information can be found online in the Supporting Information section. Graphical representation depicts a national innovation‐driven STI ecosystem. The national savings (income capital, K) of the population and human capital (H)—alongside labour parameters (L)—are comprised of intellectual, social, and infrastructural capital, which are interrelated with the GDP growth (Y) of the country. The assessment would involve analysing both backward and forward linkages between input and output. This analysis represents a trade‐off within an optimisation loop concerning total factor productivity (TFP), which is significantly influenced by innovative technologies within a semiendogenous production function.

## Data Availability

Data supporting the findings of this study are available in the Supporting Information associated with this article.
