# Supplementary figures and images for: Return on Investment (ROI) for Continuous Research and Development (R&D) Funding for Innovation‐Driven Medical Research: From Knowledge to Policy
Source: Biomed Res Int. 2026 Apr 27;2026:2544260. doi: 10.1155/bmri/2544260 (PMC13112590; doi:10.1155/bmri/2544260)

# Innovation driven -STI Ecosystem (Semi endogenous)

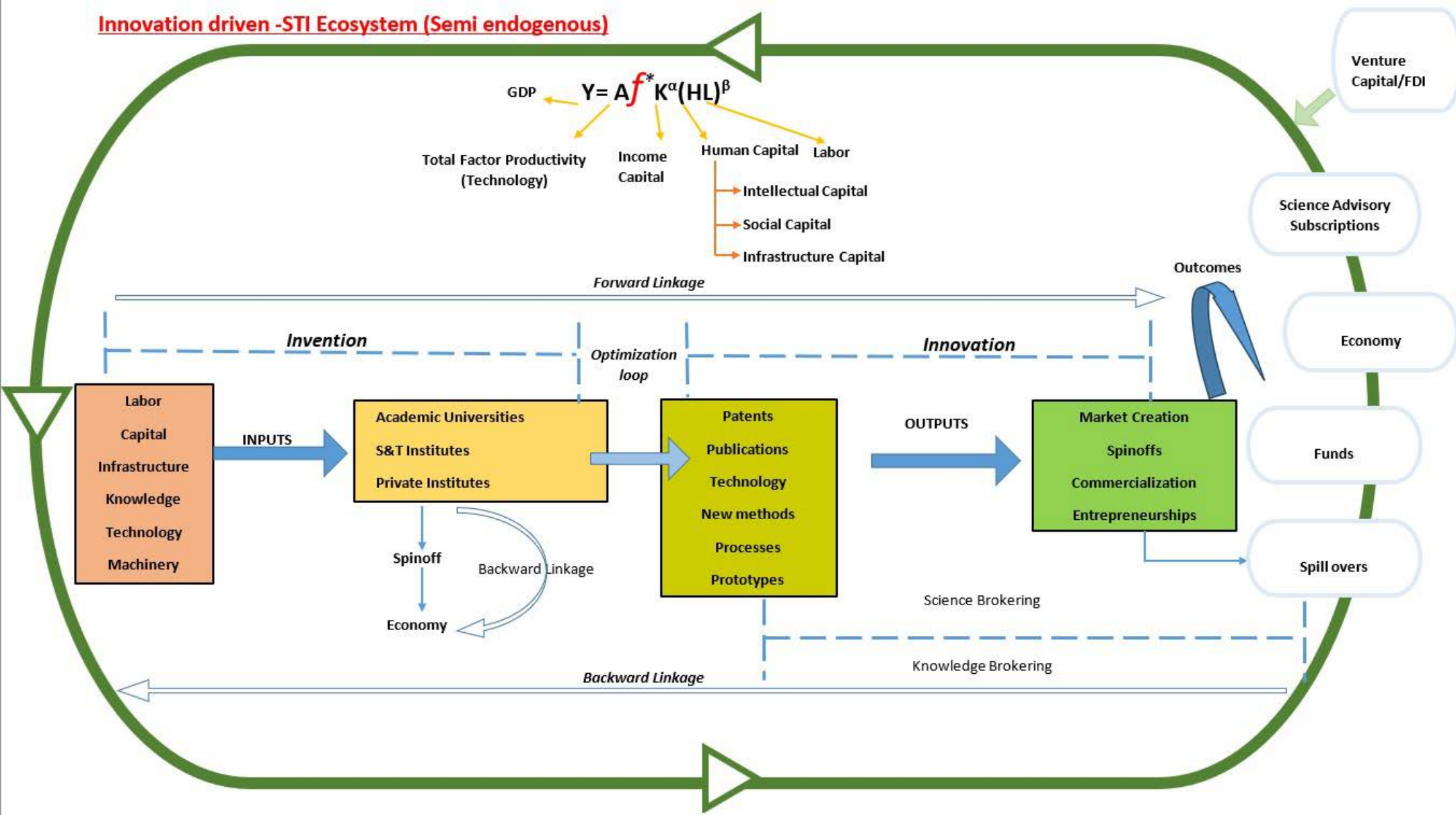

Supplement: Supplementary file 1 — Supporting Information Additional supporting information can be found online in the Supporting Information section. Graphical representation depicts a national innovation‐driven STI ecosystem. The national savings (income capital, K) of the population and human capital (H)—alongside labour parameters (L)—are comprised of intellectual, social, and infrastructural capital, which are interrelated with the GDP growth (Y) of the country. The assessment would involve analysing both backward and forward linkages between input and output. This analysis represents a trade‐off within an optimisation loop concerning total factor productivity (TFP), which is significantly influenced by innovative technologies within a semiendogenous production function. [file BMRI-2026-2544260-s001.pdf]
